# Elevated IL-17A level is associated with poor overall survival following immune checkpoint inhibitors combined with targeted therapy in hepatocellular carcinoma with hyperbilirubinemia

**DOI:** 10.3389/fimmu.2026.1791538

**Published:** 2026-04-07

**Authors:** Jianing Wang, Shida Pan, Chuyue Xiong, Jiahe Tian, Yilin Wang, Yingying Yu, Siyu Wang, Yingjuan Shen, Luo Yang, Xiaomeng Liu, Junqing Luan, Mengdie Jia, Xuanxuan Duan, Fusheng Wang, Fanping Meng

**Affiliations:** 1 302 Clinical Medical School, Peking University, Beijing, China; 2 Senior Department of Infectious Diseases, The Fifth Medical Center of Chinese People's Liberation Army (PLA) General Hospital, Beijing, China; 3 Department of Gastroenterology Endoscopy Center, Qilu Hospital (Qingdao) of Shandong University, Qingdao, Shandong, China; 4 The First Affiliated Hospital of University of Science and Technology of China (USTC), Division of Life Sciences and Medicine, University of Science and Technology of China, Hefei, China; 5 Chinese People's Liberation Army (PLA) Medical School, Beijing, China

**Keywords:** hepatocellular carcinoma, hyperbilirubinemia, prognosis, proteomics, survival

## Abstract

**Background:**

Combination therapy with programmed death-1 (PD-1) inhibitors and targeted therapy is a first-line treatment for advanced hepatocellular carcinoma (HCC). Patients with hyperbilirubinemia (total bilirubin > upper limit of normal) are often excluded from such regimens. This study aimed to evaluate the efficacy and safety of PD-1 inhibitor plus targeted therapy in this patient population and to identify plasma protein biomarkers associated with prognosis.

**Methods:**

We enrolled 201 patients with advanced HCC who received PD-1 inhibitor plus targeted therapy. After propensity score matching (1:1), 60 pairs of patients with hyperbilirubinemia and normal bilirubin were compared in terms of objective response rate (ORR), disease control rate (DCR), and overall survival (OS). Baseline plasma samples from 72 patients (28 with hyperbilirubinemia, 44 with normal bilirubin) were analyzed using Olink proteomics to quantify 92 inflammatory proteins. Differentially expressed proteins were identified, and the functional pathways were examined. The prognostic value of key proteins was assessed using Cox regression and Kaplan-Meier survival analysis.

**Results:**

The combination therapy was well tolerated in the hyperbilirubinemia group, with no increase in severe hepatotoxicity. ORR (7.0% vs. 20.7%, P = 0.043) and DCR (50.9% vs. 72.4%, P = 0.022) were significantly lower in the hyperbilirubinemia group. Median OS was significantly shorter in the hyperbilirubinemia group (HR = 0.54, 95% CI 0.35–0.84, P = 0.0065). Proteomic analysis revealed 36 differentially expressed proteins. Univariate Cox regression indicated that elevated plasma IL-17A was significantly associated with poorer OS in the hyperbilirubinemia subset (HR = 1.63, 95% CI 1.04–2.55, P = 0.033). Further stratification by median IL-17A levels showed that hyperbilirubinemia patients with high IL-17A had significantly worse OS (HR = 2.89, 95% CI 1.03–8.07, P = 0.035), whereas IL-17A levels had no significant impact in patients with normal bilirubin (P = 0.870). A formal interaction test confirmed that the detrimental effect of high IL-17A was amplified in the presence of hyperbilirubinemia (HR = 2.72, 95% CI 1.28–5.75, P = 0.009).

**Conclusion:**

PD-1 inhibitor combined with targeted therapy is safe and feasible in advanced HCC patients with hyperbilirubinemia, although efficacy is inferior to that in patients with normal bilirubin. Elevated plasma IL-17A may serve as a biomarker for poor prognosis and a potential therapeutic target in this population.

## Introduction

Liver cancer represents a major global health challenge and is a leading cause of cancer-related mortality (1). Among these, HCC is the most prevalent subtype of primary liver cancer (2). HCC is currently the sixth most common malignancy and the third leading cause of cancer-related death worldwide (3, 4), with a global 5-year survival rate of less than 20% (5). Owing to the absence of specific early symptoms, the majority of patients are diagnosed at an advanced stage, which confers a poor prognosis (6). In recent years, with the development and application of novel drugs, systemic therapies, particularly molecular targeted therapy and immunotherapy, have achieved major breakthroughs in treating intermediate and advanced HCC (7). Among various cancer immunotherapies, immune checkpoint inhibitors (ICIs) targeting molecules such as programmed death-1 (PD-1) and cytotoxic T lymphocyte-associated protein-4 (CTLA-4) have become a promising approach (8, 9). Current research focuses on combination strategies of ICIs with tyrosine kinase inhibitors (TKIs), whose synergistic effects have demonstrated significant advantages in clinical trials (10, 11).

Bilirubin is primarily derived from heme released during the breakdown of senescent red blood cells and is widely regarded as an important diagnostic marker for liver and blood diseases (12). Hyperbilirubinemia, defined as a TBil level exceeding 17.1 μ7.1eL, is relatively common in HCC patients (12, 13). Jaundice occurs in 19%-40% of HCC patients at diagnosis, typically in advanced stages (14). However, given that combination therapy with PD-1 inhibitors and targeted therapy may potentially lead to liver failure, patients with elevated TBil were historically considered unsuitable for such treatment. Our previous study suggested that PD-1 inhibitor plus targeted combination therapy could be safely administered to patients with hyperbilirubinemia (15).

In recent years, advances in proteomic technologies have enabled in-depth investigation of key proteins within the tumor microenvironment. Plasma proteomics has been widely applied in researching biomarkers for tumor treatment efficacy (16). By comparing baseline differential proteins between HCC patients with hyperbilirubinemia and those with normal TBil, molecular pathways and key regulatory factors associated with hyperbilirubinemia can be revealed. This study aims to identify differential proteins related to the efficacy and prognosis of targeted immunotherapy in HCC patients with hyperbilirubinemia through proteomic analysis and further explore their mechanisms in HCC development, providing new targets for individualized treatment.

## Materials and methods

### Participants

From June 2020 to August 2024, 201 patients with advanced HCC receiving PD-1 inhibitor and targeted combination therapy at the Department of Biological Injury of the Fifth Medical Center of the PLA General Hospital were included, Among them, 131 patients had hyperbilirubinemia at baseline. According to PD-1 inhibitor prescribing information and NCCN Clinical Practice Guidelines (17–19), these patients were not recommended for ICI therapy. Before starting ICI and targeted therapy, these patients received only conservative supportive care. Patients underwent comprehensive assessment by a multidisciplinary team before treatment, and bilirubin levels and liver function were closely monitored during treatment. To control for confounding bias, eligible patients with hyperbilirubinemia were matched 1:1 with patients with baseline normal TBIL (TBIL ≤17.1 μmol/L) using propensity score matching (PSM). Matching variables included age, sex, BCLC stage, Immunotherapy, Combination targeted therapy, alpha-fetoprotein (AFP) level, etc. The matching was performed with a caliper width of 0.2 times the standard deviation of the logit of the propensity score, as recommended by Austin (2011) to balance matching accuracy and sample size preservation. Patients with missing data for any matching variable were excluded from the analysis; the proportion of missingness was low (<5%). This resulted in matched hyperbilirubinemia (n=60) and normal bilirubin (n=60) groups for clinical efficacy comparison. From the above cohort receiving combination therapy, 72 patients with available baseline plasma samples were further selected and divided into hyperbilirubinemia (n=28) and normal bilirubin (n=44) subgroups for subsequent proteomic analysis.

### Inclusion and exclusion criteria

Inclusion criteria were as follows: age ≥18 years; histologically/radiologically confirmed advanced HCC; patients receiving at least one cycle of ICI combined with targeted therapy; baseline TBil level meeting the criteria for the hyperbilirubinemia group (>17.1 μmol/L) or normal group (≤17.1 μmol/L); baseline prothrombin activity (PTA) >40%; at least one measurable target lesion according to the modified Response Evaluation Criteria in Solid Tumors (mRECIST) (20, 21). Exclusion criteria were as follows: concurrent other primary tumors; severe coagulation dysfunction; concurrent severe cardiac or renal insufficiency; and poorly controlled diabetes and hypertension. This study was approved by the Ethics Committee of the Fifth Medical Center of the Chinese PLA General Hospital (IRB No. KY-2023-7-46-1).

### Drug administration

PD-1 inhibitors: Sintilimab (Innovent Biologics, China), Camrelizumab (Hengrui Medicine, China), and Tislelizumab (BeiGene, China) were administered at a fixed dose of 200 mg every three weeks; Toripalimab (Shanghai Junshi Biosciences, China) was administered at 240 mg every three weeks. The PD-1/CTLA-4 bispecific antibody, Cadonilimab (Akeso Biopharma, China),was administered at 250 mg every three weeks. Sorafenib (Bayer, Germany) was administered at 400 mg twice daily; Lenvatinib (Eisai, Japan) was administered at 8 mg or 12 mg once daily based on body weight; Bevacizumab (Qilu Pharmaceutical, China) was administered at 7.5 mg/kg every three weeks. Specific drug choices were based on the attending physician’s decision and patient condition.

### Proteomic analysis of soluble factors in plasma

Plasma levels of 92 immune-related proteins (IRPs) were quantified using the Olink multiplex proximity extension assay (PEA) Inflammation panel (Olink Bioscience AB, Sweden). The PEA technique employs pairs of oligonucleotide-labeled antibodies that bind to their respective target proteins. When bound in close proximity, the oligonucleotides hybridize and are extended by a DNA polymerase, generating a unique DNA barcode that is subsequently amplified and quantified via real-time PCR. Protein expression levels are reported as Normalized Protein Expression (NPX) values, an arbitrary, log2trary, lyides unit. Rigorous quality control was implemented throughout the experimental workflow. Prior to analysis, all plasma samples were assessed for hemolysis and lipid content. From the overall cohort of 120 patients, 72 patients with available baseline plasma samples were selected for proteomic analysis. All 72 samples passed initial quality checks and were processed in a single batch to minimize interized variability. Each assay plate included internal controls (incubation, extension, and amplification controls) to monitor the efficiency of key procedural steps. Inter−plate controls (IPCs) were used to normalize data across different plates. Further quality filtering was applied at both the sample and protein levels. At the sample level, samples with an overall detection rate below 75% or with NPX values outside the pre-defined limit of detection (LOD) for more than 50% of the assays were excluded; all 72 selected samples met the quality criteria. At the protein level, 19 proteins with more than 90% of their measurements below the detection limit were filtered out from downstream analysis. Consequently, the final dataset included 72 samples and 73 reliably detected IRPs for subsequent statistical and functional analyses.

### Statistical analysis

Statistical analysis was performed using R software (version 4.3.1). Categorical variables are expressed as counts and percentages (%). Normality was assessed using the Kolmogorov-Smirnov test. Continuous variables are reported as mean ± standard deviation. Based on data distribution, Student’s t-test or Mann-Whitney U test was used for comparing continuous variable groups, while Chi-square test or Fisher’s exact test was used for analyzing categorical outcomes. To minimize selection bias and potential confounders, propensity score matching (PSM) analysis was employed. Statistical significance was defined as a Benjamini-Hochberg adjusted P-value (adj.P.Val) < 0.05, and a biological significance threshold was set at an absolute log2 fold-change (|log2FC|) > 0.5. Survival analysis was performed using the Kaplan-Meier method and log-rank tests. Overall survival was measured from the initiation of treatment to death. The impact of survival prognostic factors was analyzed using univariate and multivariate Cox regression models. Statistical significance was set at a two-sided alpha value of 0.05.

## Results

### Baseline characteristics of patient

After PSM matching, there were 60 patients each in the hyperbilirubinemia and normal bilirubin groups. No statistically significant differences were observed between the two groups in key baseline characteristics including age, sex, BCLC stage, AFP level, and treatment regimen (all P>0.05). By design, significant differences remained in total bilirubin (TBIL) levels, which is a component of both the Child-Pugh and mALBI grading systems. Consequently, the hyperbilirubinemia group had significantly worse Child-Pugh and mALBI grades (all P < 0.05), reflecting greater severity of underlying liver dysfunction. The complete baseline data are summarized in **Table 1**.

**Table 1:** Baseline characteristics of patients after propensity score matching.

Variables	Total	Hyperbilirubinemia group	Normal bilirubin group	P value
	*N=120*	*N=60*	*N=60*	
Age	56.96 ± 11.18	55.04 ± 10.76	58.18 ± 11.39	0.975
Sex				0.765
Male	104 (86.7%)	52 (86.7%)	52 (86.7%)	
Female	16 (13.3%)	8 (13.3%)	8 (13.3%)	
BCLC stage				0.236
A	10 (8.3%)	2(3.3%)	8 (13.3%)	
B	36 (30.0%)	22 (37.9%)	14 (23.3%)	
C(M)	24 (20.0%)	11 (18.3%)	13 (21.7%)	
C(PVTT)	46 (38.3%)	23 (38.3%)	23 (38.3%)	
D	4 (3.3%)	2 (3.3%)	2 (3.3%)	
Child-Pugh stage				0.008
A	42(35.0%)	22(36.7%)	20(33.3%)	
B	74(61.7%)	35(58.3%)	39(65.0%)	
C	4(3.3%)	3(5.0%)	1(1.7%)	
mALBI				0.016
1	26(21.7%)	8(13.3%)	18(30.0%)	
2	79(65.8%)	38(63.3%)	41(68.3%)	
3	15(12.5%)	14(23.3%)	1(1.7%)	
Baseline of PA (%)	77.70(67.00, 93.60)	73.15(62.50, 90.80)	84.25(69.60, 97.60)	0.906
TBIL (μmol/L)	16.95 [13.45;42.12]	42.65 [23.10;169.10]	13.40 [10.50;15.07]	<0.001
AFP				0.826
<400ng/mL	80 (69.6%)	40 (67.8%)	40 (71.4%)	
≥400ng/mL	35 (30.4%)	19 (32.2%)	16 (28.6%)	
ALB(g/L)	35.00 [31.00;39.00]	34.00 [29.75;37.00]	36.00 [32.00;39.00]	0.056
CRP(ng/mL)	6.40 [2.65;27.12]	9.90 [3.20;28.72]	3.80 [1.65;23.70]	0.064
ALT(U/L)	38.50 [25.00;58.00]	46.50 [25.00;71.75]	33.50 [25.50;53.25]	0.142
AST(U/L)	56.00 [41.50;91.00]	66.00 [42.75;110.00]	50.00 [39.25;72.25]	0.060
Immunotherapy				0.254
Sintilimab	77(64.2%)	35(58.3%)	38(63.3%)	
Camrelizumab	18(15.0%)	7(11.7%)	11(18.3%)	
Tislelizumab	14(11.7%)	10(16.7%)	5(8.3%)	
Cadonilimab	9(5.8%)	7(11.7%)	3(5.0%)	
Toripalimab	4(3.3%)	1(1.6%)	3(5.0%)	
Combination targeted therapy				0.281
Lenvatinib	93(77.5%)	43(71.6%)	50(83.3%)	
Bevacizumab	12(10.0%)	7(11.7%)	5(8.3%)	
Sorafenib	11(9.2%)	4(6.7%)	7(11.7%)	
Regorafenib	1(0.8%)	1(1.6%)	0(0%)	
Apatinib	3(2.5%)	1(1.6%)	2(3.2%)	

Continuous variables are presented as mean ± SD or median (interquartile ranges). TBIL, total bilirubin; AFP, alpha-fetoprotein; PT, prothrombin time; CRP, C-reactive protein; ALT, alanine aminotransferase; AST, aspartate aminotransferase.

### Antitumor efficacy and survival outcomes

**Figure 1:**
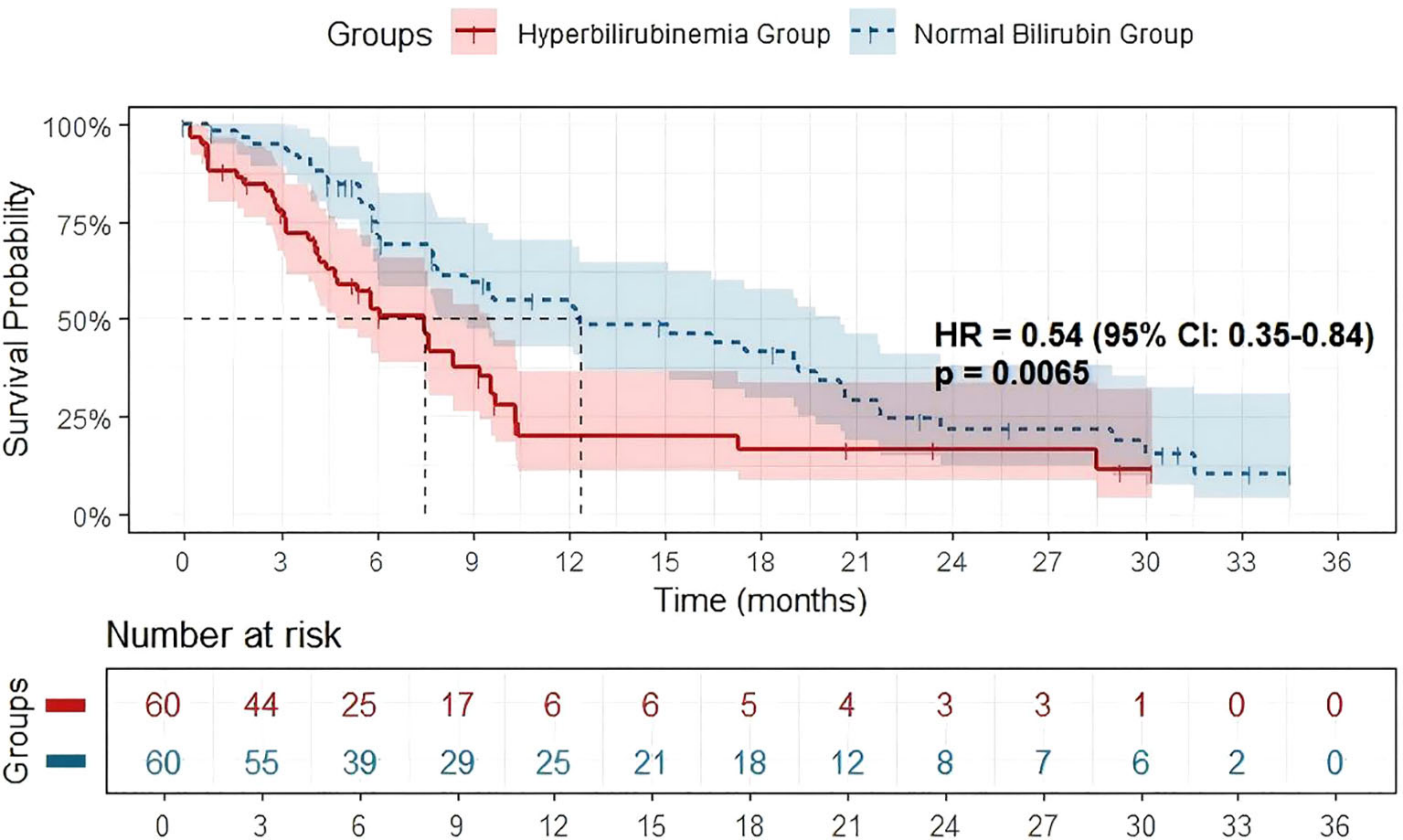
Kaplan-Meier survival analysis comparing overall survival between advanced HCC patients with hyperbilirubinemia and those with normal bilirubin receiving anti-PD-1 and targeted combination therapy. The median OS was significantly longer in the normal bilirubin group (HR = 0.54 (95% CI: 0.35-0.84), p = 0.0065).

As of September 1, 2024, tumor responses were evaluated according to mRECIST. While 60 patients were matched in each group after PSM, a small number were not evaluable for radiographic response at the first scheduled assessment (within approximately 3 months) due to early loss to follow-up. Consequently, the efficacy-evaluable population comprised 57 patients in the hyperbilirubinemia group and 58 in the normal bilirubin group. Less than 20% of patients were lost to follow-up in OS. In this evaluable population, ORR was significantly lower in the hyperbilirubinemia group (7.0% vs. 20.7%, P = 0.043). DCR was significantly lower in the hyperbilirubinemia group (50.9% vs. 72.4%, P = 0.022). **Table 2**. Kaplan-Meier survival curves showed that median OS was significantly longer in the normal bilirubin group compared to the hyperbilirubinemia group (HR = 0.54, 95% CI 0.35-0.84, P = 0.0065). This indicates that TBIL level is a significant factor affecting patient prognosis (**Figure 1**).

**Table 2:** Comparison of tumor treatment response between the two groups (mRECIST).

Tumor response (mRECIST)	Hyperbilirubinemia group (N = 57)	Normal bilirubin group (N = 58)	P value
CR	0 (0%)	3 (5.2%)	
PR	4 (7.0%)	9 (15.5%)	
SD	25 (43.9%)	30 (51.7%)	
PD+death	28 (49.1%)	16 (27.6%)	
ORR(CR+PR)	4 (7.0%)	12 (20.7%)	0.043*
DCR(CR+PR+SD)	29 (50.9%)	42 (72.4%)	0.022*

mRECIST, modified Response Evaluation Criteria in Solid Tumors; CR, complete response; PR, partial response; SD, stable disease; PD, progressive disease; OR, objective response; DC, disease control. *<0.05.

### Screening results for plasma differentially expressed proteins between groups

**Figure 2:**
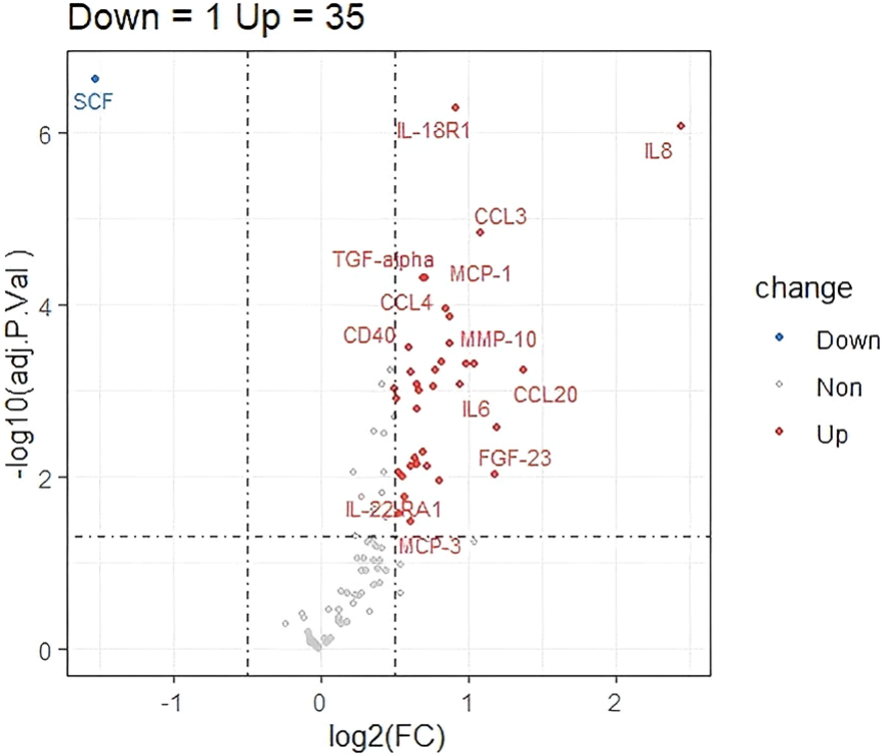
Volcano plot of differentially expressed proteins. A total of 36 proteins were significantly dysregulated (35 up-regulated and 1 down-regulated, P < 0.05 and |log2FC| ≥ 0.5) in the hyperbilirubinemia group compared to the normal bilirubin group.

**Figure 3:**
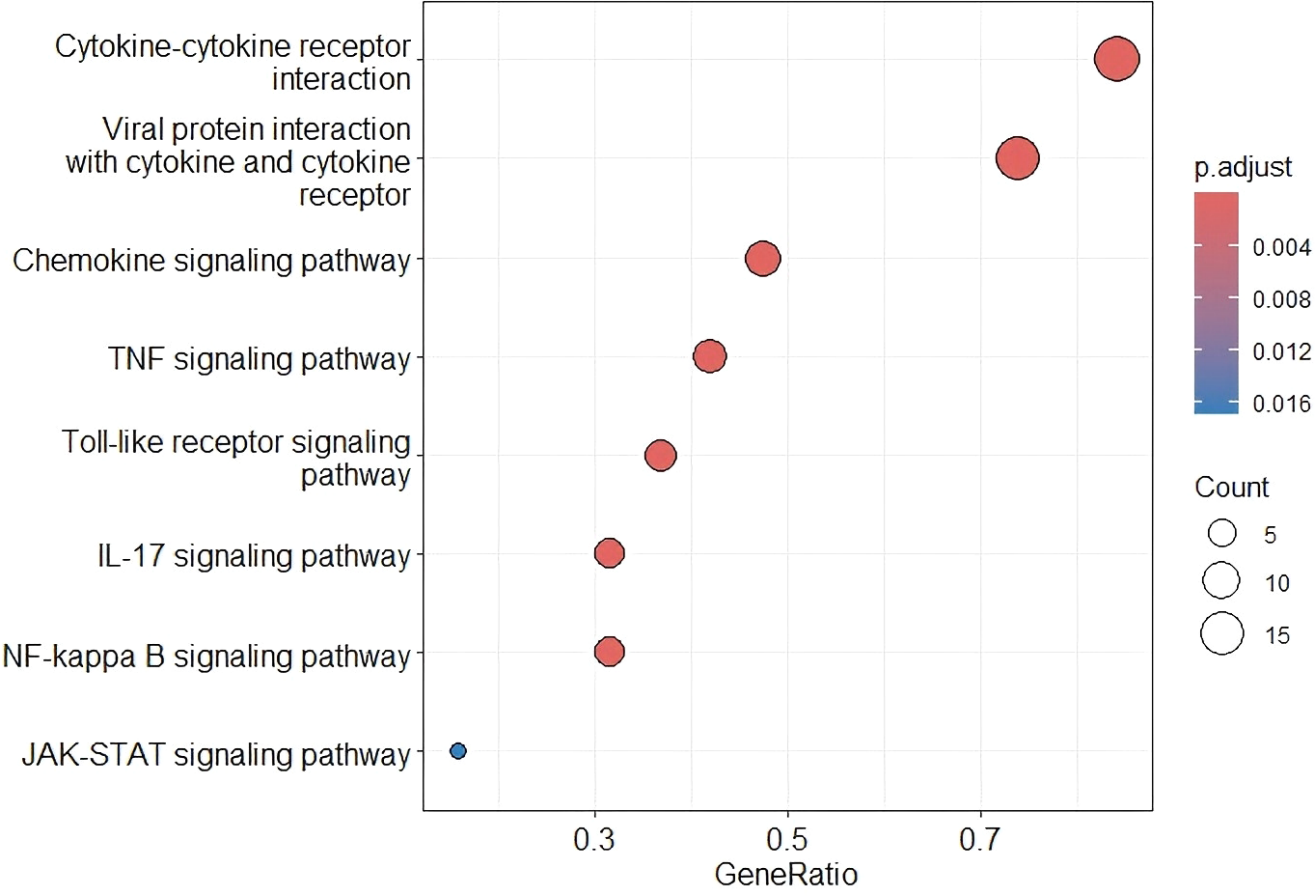
KEGG pathway enrichment analysis of the differentially expressed proteins.​ The most significantly enriched pathways are shown, highlighting the central role of cytokine-cytokine receptor interaction and IL-17 signaling pathway.

Key baseline characteristics between the two groups shown in **Supplementary Table 1**. In the protein expression profile data from HCC patients with hyperbilirubinemia and those with normal TBil, a total of 36 differentially expressed proteins were screened using thresholds of P value <0.05 and |log2(FoldChange)| ≥log2 In the hyperbilirubinemia HCC group, 35 proteins were significantly up-regulated and 1 protein was significantly down-regulated(**Supplementary Table 2**).The expression patterns of the differential proteins were visualized via volcano plot, intuitively reflecting the differential expression patterns between the two sample groups (**Figure 2**). To understand the functional differences of the differentially expressed proteins between the two patient groups, KEGG pathway enrichment analysis was performed. Using an adjusted P value (P.adjust) <0.01 as the significance threshold, 29 significantly enriched pathways were identified. The bubble plot clearly shows the most significantly enriched pathways, including “Cytokine-cytokine receptor interaction,” “Viral protein interaction with cytokine and cytokine receptor,” “TNF signaling pathway,” “Chemokine signaling pathway,” “Toll-like receptor signaling pathway,” “IL-17 signaling pathway,” “NF-kappa B signaling pathway,” and “JAK-STAT signaling pathway” (**Figure 3**). The significant enrichment of these pathways provides important clues to the underlying biological mechanisms in HCC patients with hyperbilirubinemia.

### Relationship between differential protein expression and overall survival in HCC patients with hyperbilirubinemia

**Figure 4:**
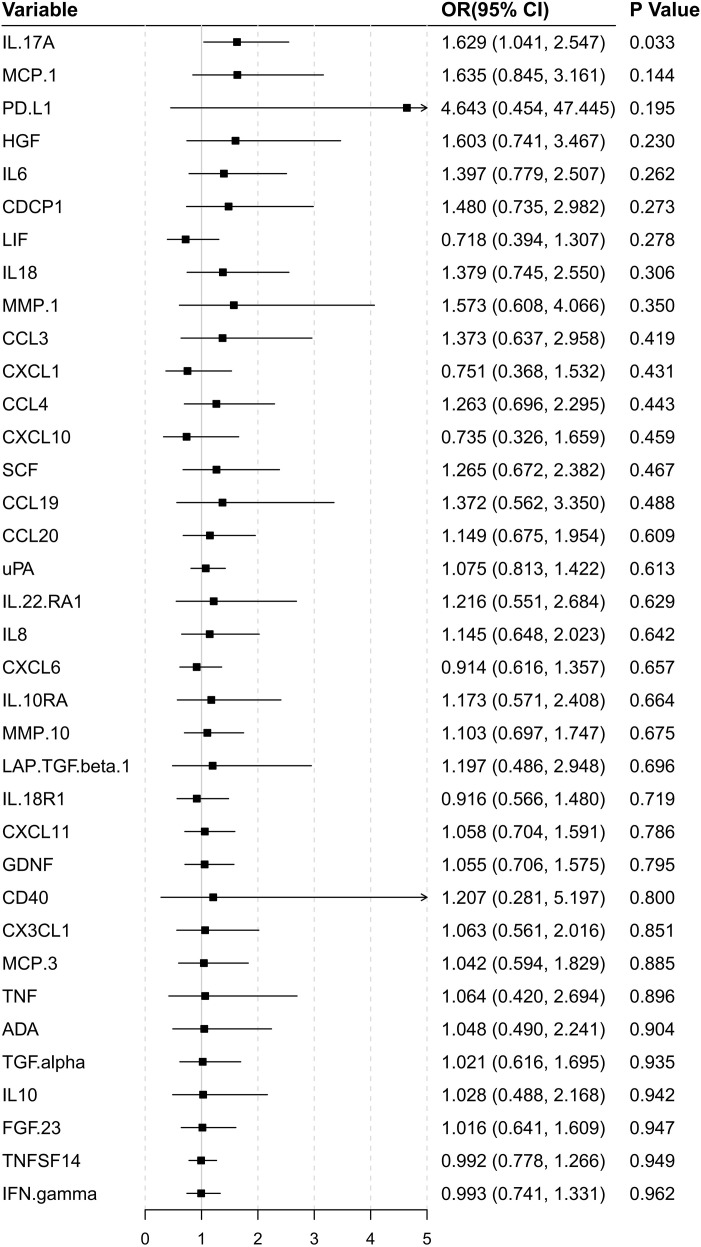
Forest plot of univariate Cox regression analysis (HR = 1.63, 95% CI 1.04–2.55, P = 0.033).

In the hyperbilirubinemia subset, univariate Cox regression was performed on the 36 differentially expressed proteins. To control for multiple testing, we applied the Benjamini-Hochberg procedure across all 36 proteins. After adjustment, IL-17A remained the only protein significantly associated with overall survival (HR = 1.63, 95% CI 1.04–2.55, adjusted P = 0.033) (**Figure 4**), indicating that its prognostic value is robust to multiple testing correction and independent of baseline clinicopathological characteristics.

To examine whether this association is context-specific, we stratified the entire cohort into four groups using bilirubin status and the median IL-17A level calculated within each bilirubin stratum: (1) hyperbilirubinemia & low IL-17A, (2) hyperbilirubinemia & high IL-17A, (3) normal bilirubin & low IL-17A, and (4) normal bilirubin & high IL-17A.

**Figure 5:**
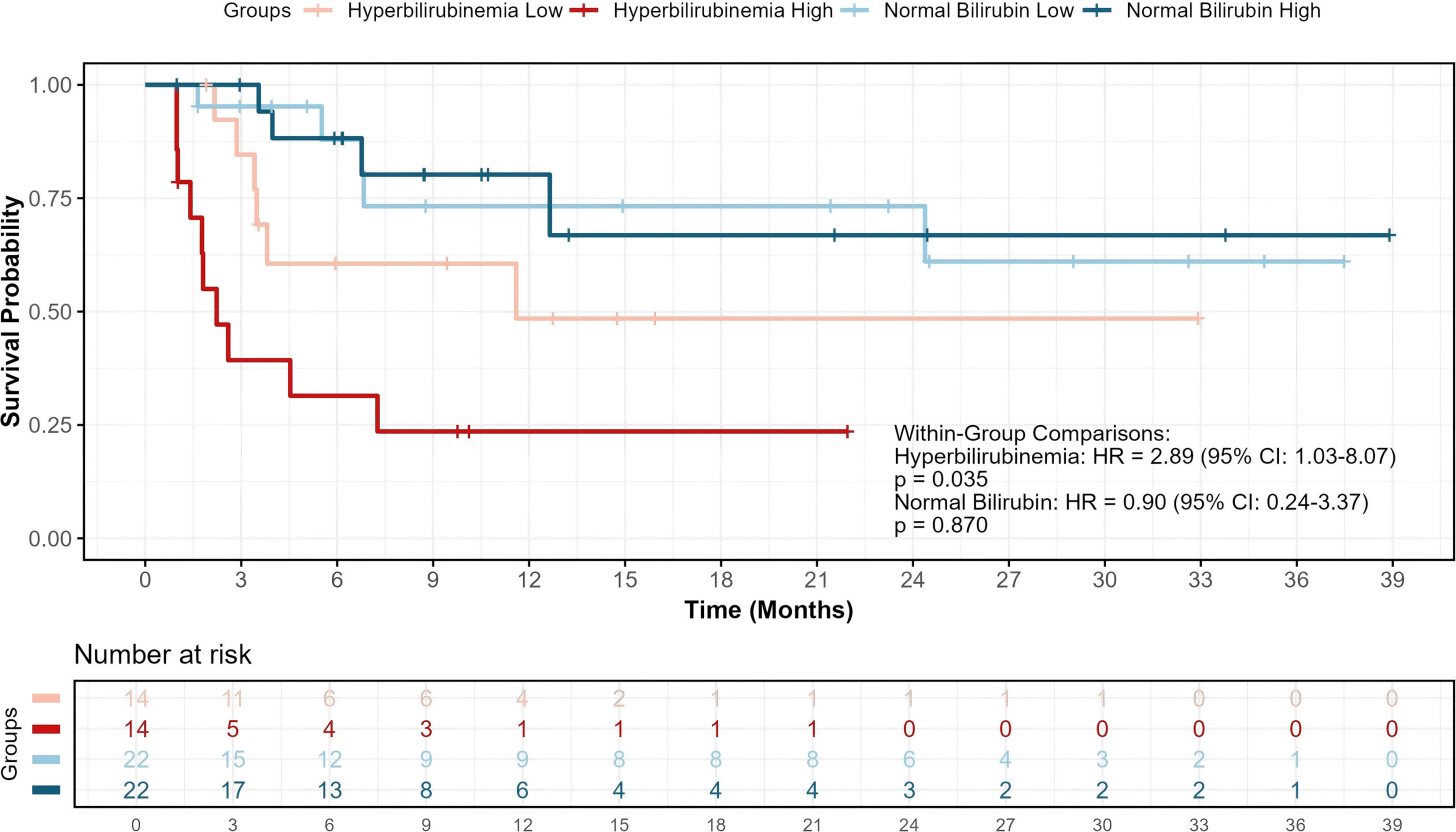
Overall survival stratified by IL-17A levels within bilirubin-based subgroups. Kaplan-Meier curves depict overall survival in patients with advanced hepatocellular carcinoma receiving PD-1 inhibitor plus targeted therapy, stratified by baseline bilirubin status and plasma IL-17A levels (high vs. low, based on the median within each bilirubin stratum). Within the hyperbilirubinemia cohort, high IL-17A expression is significantly associated with inferior survival compared to low IL-17A (HR = 2.89, 95% CI: 1.03–8.07, P = 0.035). No significant survival difference was observed between high and low IL-17A groups in patients with normal bilirubin (HR = 0.90, 95% CI: 0.24–3.37, P = 0.870).

Kaplan-Meier analysis revealed significant heterogeneity across the four groups (**Figure 5**). Pairwise comparisons showed that patients with hyperbilirubinemia and high IL-17A expression had the shortest survival, differing significantly from each of the other three groups (all P < 0.05). In contrast, within the normal-bilirubin stratum, high versus low IL-17A expression did not confer a statistically significant survival difference (HR = 0.90, 95% CI 0.24–3.37, P = 0.870). Conversely, among hyperbilirubinemic patients, high IL-17A expression was associated with markedly poorer prognosis (HR = 2.89, 95% CI 1.03–8.07, P = 0.035) (**Figure 5**).

**Figure 6:**
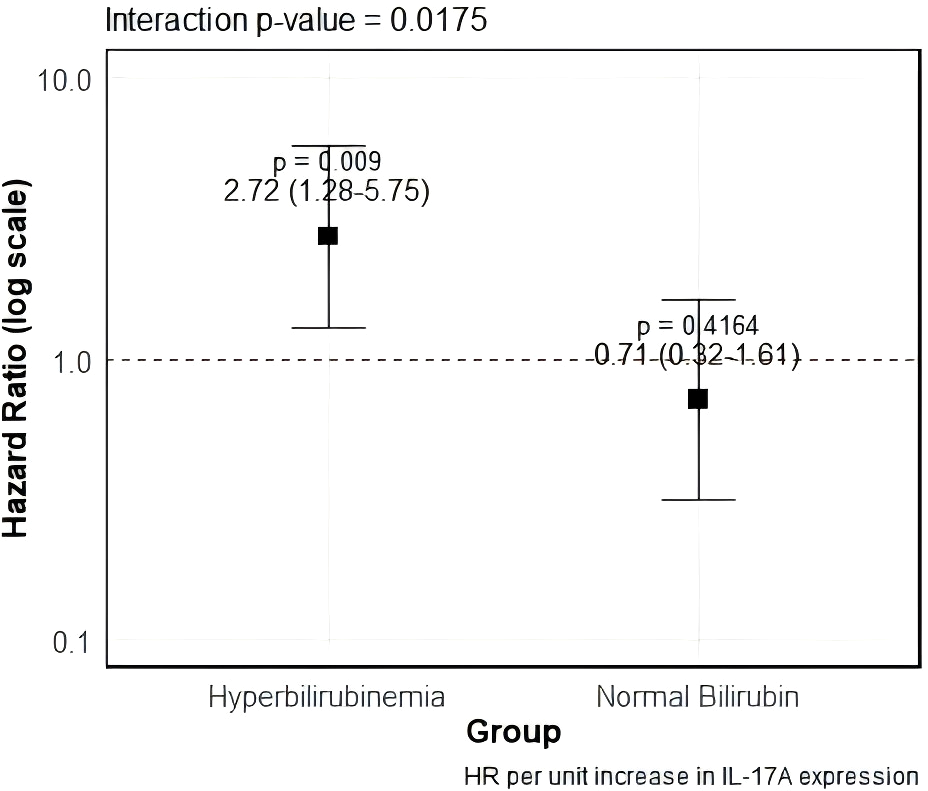
Interaction between hyperbilirubinemia status and elevated IL-17A on overall survival.

To formally test for interaction, we introduced a product term (hyperbilirubinemia status × IL-17A high/low) into a Cox model. The interaction term was statistically significant (HR for interaction = 2.72, 95% CI 1.28,5.75, P interaction = 0.009), indicating that the detrimental effect of high IL-17A on survival was significantly amplified in patients with hyperbilirubinemia (**Figure 6**).

Collectively, these data identify IL-17A as a context-specific prognostic biomarker in HCC patients receiving PD-1 inhibitor plus targeted therapy. Our findings suggest that elevated IL-17A expression, particularly when combined with hyperbilirubinemia, may reflect an inflammatory tumor microenvironment associated with aggressive disease biology and poor therapeutic response. Further functional studies are warranted to elucidate the mechanistic role of IL-17A in this high-risk patient population.

## Discussion

In China, HCC patients face a worse prognosis, with a 5-year survival rate as low as 12% (22). Despite significant advances in diagnostic techniques and treatment methods, HCC patients’ early symptoms are non-specific, leading to diagnosis at intermediate or advanced stages for most patients, which greatly limits treatment options and survival rates (23, 24). Furthermore, HCC patients often have multiple complications such as cirrhosis, ascites, and hyperbilirubinemia, which further exacerbate the disease complexity and treatment difficulty. Hyperbilirubinemia is relatively common in HCC patients and may affect tumor biology and patient prognosis through various mechanisms (25). However, the mechanisms underlying hyperbilirubinemia’s role in HCC remain unclear, and related research is limited. This study, using proteomic technology, systematically compared for the first time the baseline plasma protein profiles between advanced HCC patients with hyperbilirubinemia and those with normal TBil, and on this basis revealed the independent prognostic value of the inflammatory cytokine IL-17A in this high-risk population.

One of the most important findings is that HCC patients with hyperbilirubinemia exhibit a strong inflammatory response state in their baseline plasma. Globally, up to 20% of cancer cases are associated with chronic inflammation or chronic infection, particularly HCC related to hepatitis infection (4, 26). Inflammation modulates the tumor microenvironment by altering the balance of cytokines, chemokines, matrix-degrading enzymes, transcription factors, and reactive oxygen species (ROS), thereby influencing tumorigenesis at the molecular level (27). Subsequent univariate Cox regression analysis confirmed that IL-17A is a significant factor associated with poor prognosis in hyperbilirubinemia HCC patients. Survival analysis further showed that within the hyperbilirubinemia group, patients with high IL-17A expression had a significantly shortened median overall survival. This finding holds important clinical significance as it is the first to link IL-17A expression levels with long-term survival in this specific high-risk population. The underlying mechanism may involve multiple layers: First, studies by Li et al. have confirmed that IL-17A can directly induce the expression of molecules like MMP2 and MMP9 in HCC cells and the microenvironment by activating the NF-κB pathway, promoting tumor invasion (28). Second, as shown in this study and previous reports, IL-17A can synergize with other inflammatory factors and upregulate VEGF, disrupting vascular homeostasis and promoting tumor angiogenesis (29). Finally, and crucially, IL-17A shapes a profoundly immunosuppressive microenvironment by recruiting and utilizing myeloid-derived suppressor cells (MDSCs) to regulate the Treg/Th17 balance (30), and by inducing PD-L1 expression on tumor cells (31), which may be a key mechanism leading to resistance to ICI therapy. Based on our findings and prior mechanistic studies, we hypothesize a potential interplay between hyperbilirubinemia and IL-17A: persistent liver injury and inflammation associated with elevated bilirubin may promote IL-17A overexpression, which in turn could contribute to an immunosuppressive microenvironment and resistance to immunotherapy. However, this hypothesis-generating observation requires validation in prospective cohorts and functional studies to establish causality.

The findings of this study provide a new perspective for the clinical management of advanced HCC patients with hyperbilirubinemia. On one hand, Plasma IL-17A levels hold promise as a novel biomarker for risk stratification and prognosis assessment in this population, helping to identify patients requiring more aggressive interventions. On the other hand, IL-17A itself becomes a highly potential therapeutic target (32–35). Currently, various immune checkpoint inhibitors are widely promoted for cancer treatment (36, 37). Notably, in clinical practice, IL-17A pathway activity is closely associated with adverse reactions to ICI therapy. Basic research and preliminary clinical explorations in other cancers (such as lung cancer, colorectal cancer) suggest that targeting the IL-17A signaling pathway (e.g., using IL-17A neutralizing antibodies) may reverse tumor-associated immunosuppression and produce synergistic antitumor effects with existing PD-1/PD-L1 inhibitors (35, 38). For advanced HCC patients with hyperbilirubinemia, who currently have limited treatment options and poor efficacy, exploring combination strategies like “ICIs + targeted therapy + anti-IL-17A” may be a breakthrough path worthy of in-depth investigation.

However, this study also has certain limitations. First, it is a single-center retrospective study with a relatively small sample size, requiring validation by multi-center prospective cohorts in the future. Second, the study is based on plasma proteomics, which reflects systemic status but does not directly reveal inflammatory cell infiltration and factor expression within the local tumor microenvironment. Future validation using tumor tissue would substantially strengthen the biological plausibility of our findings and help elucidate the mechanistic role of IL-17A in this context. Third, while we observed no increase in severe hepatotoxicity, suggesting acceptable hepatic safety in this population, our assessment of safety was primarily focused on liver-related events. A comprehensive safety profile, including the full spectrum of immune-related adverse events (irAEs) affecting other organ systems, warrants evaluation in larger prospective cohorts with standardized AE monitoring. Furthermore, the study’s cross-sectional design limits the ability to assess changes in protein expression over time. Future research should explore temporal dynamics of protein expression by conducting repeated sampling over a longer period.

In summary, this study revealed differential proteins and their related pathways between advanced HCC patients with hyperbilirubinemia and those with normal bilirubin, and identified IL-17A as a key factor affecting the prognosis of HCC patients with hyperbilirubinemia. These findings not only provide theoretical support for a deeper understanding of the mechanisms of hyperbilirubinemia in HCC but also offer potential targets for future treatment strategies. Future research should further expand the sample size, conduct comprehensive analysis incorporating tumor tissue samples, and delve deeper into the mechanisms of action of key regulatory proteins, aiming to provide stronger support for the clinical treatment of HCC.

## Data Availability

The data used to support the findings of this study are available from the corresponding author upon reasonable request.
