# Correction: Elevated IL-17A level is associated with poor overall survival following immune checkpoint inhibitors combined with targeted therapy in hepatocellular carcinoma with hyperbilirubinemia

**DOI:** 10.3389/fimmu.2026.1857653

**Published:** 2026-04-27

**Authors:** Jianing Wang, Shida Pan, Chuyue Xiong, Jiahe Tian, Yilin Wang, Yingying Yu, Siyu Wang, Yingjuan Shen, Luo Yang, Xiaomeng Liu, Junqing Luan, Mengdie Jia, Xuanxuan Duan, Fusheng Wang, Fanping Meng

**Affiliations:** 1 302 Clinical Medical School, Peking University, Beijing, China; 2 Senior Department of Infectious Diseases, The Fifth Medical Center of Chinese People’s Liberation Army (PLA) General Hospital, Beijing, China; 3 Department of Gastroenterology Endoscopy Center, Qilu Hospital (Qingdao) of Shandong University, Qingdao, Shandong, China; 4 The First Affiliated Hospital of University of Science and Technology of China (USTC), Division of Life Sciences and Medicine, University of Science and Technology of China, Hefei, China; 5 Chinese People’s Liberation Army (PLA) Medical School, Beijing, China

**Keywords:** hepatocellular carcinoma, hyperbilirubinemia, prognosis, proteomics, survival

Affiliation “Department of Gastroenterology Endoscopy Center, Qilu Hospital (Qingdao) of Shandong University, Qingdao, Shandong, China” was omitted for author Shida Pan. This affiliation has now been added for author Shida Pan.

Author Shida Pan was erroneously assigned to affiliation “Senior Department of Infectious Diseases, The Fifth Medical Center of Chinese People’s Liberation Army (PLA) General Hospital, Beijing, China”. This affiliation has now been removed for author Shida Pan.

Author Chuyue Xiong was erroneously omitted as equal contributing author.

The original version of this article has been updated.

