# Supplementary material for: Elevated IL-17A level is associated with poor overall survival following immune checkpoint inhibitors combined with targeted therapy in hepatocellular carcinoma with hyperbilirubinemia
Source: Front Immunol. 2026 Apr 7;17:1791538. doi: 10.3389/fimmu.2026.1791538 (PMC13095688; doi:10.3389/fimmu.2026.1791538)
Supplement: Supplementary file 1 [file Table1.docx]

**Table S1. Baseline characteristics of the proteomics analysis cohort (n=72).**

|  | **Total** | **Hyperbilirubinemia Group** | **Normal Bilirubin Group** | **P value** |
| --- | --- | --- | --- | --- |
|  | ***N=72*** | ***N=28*** | ***N=44*** |  |
| Age | 54.33±10.89 | 55.04±10.76 | 57.15±11.85 | 0.472 |
| Sex |  |  |  | 0.973 |
| Male | 59 (81.9%) | 23 (82.1%) | 36 (81.8%) |  |
| Female | 13 (18.1%) | 5 (17.9%) | 8 (18.2%) |  |
| BCLCstage |  |  |  | 0.402 |
| A | 9 (12.5%) | 2 (7.1%) | 7 (15.9%) |  |
| B | 22 (30.6%) | 6 (21.4%) | 16 (36.4%) |  |
| C(M) | 19 (26.4%) | 9 (32.1%) | 10 (22.7%) |  |
| C(PVTT) | 20 (27.8%) | 10 (35.7%) | 10 (22.7%) |  |
| D | 2 (2.8%) | 1 (3.6%) | 1 (2.3%) |  |
| Child-Pugh stage |  |  |  | <0.001 |
| A | 30 (41.7%) | 4 (14.3%) | 26 (59.1%) |  |
| B | 38 (52.8%) | 20 (71.4%) | 18 (40.9%) |  |
| C | 4 (5.6%) | 4 (14.3%) | 0 (0%) |  |
| mALBI |  |  |  | <0.001 |
| 1 | 12 (16.7%) | 1 (3.6%) | 11 (25.0%) |  |
| 2 | 49 (68.1%) | 17 (60.7%) | 32 (72.7%) |  |
| 3 | 11 (15.3%) | 10 (35.7%) | 1 (2.3%) |  |
| Baseline of PA (%) | 67.00(58.10, 79.40) | 65.85(58.10, 72.60) | 70.85(58.08, 93.80) | 0.193 |
| TBIL (μmol/L) | 30.50 [13.45;80.30] | 135.85 [67.15;200.10] | 13.40 [10.50;17.09] | <0.001 |
| AFP |  |  |  | 0.509 |
| <400ng/mL | 52 (72.2%) | 19 (67.9%) | 33 (75.0%) |  |
| ≥400ng/mL | 20 (27.8%) | 9 (32.1%) | 11 (25.0%) |  |
| ALB(g/L) | 35.00 [31.00;39.00] | 34.00 [33.00;38.00] | 37.00 [32.00;40.00] | 0.077 |
| CRP(ng/mL) | 6.40 [2.65;27.12] | 14.90 [9.60;27.00] | 6.00 [1.75;34.05] | 0.064 |
| ALT(U/L) | 38.50 [22.00;69.00] | 44.50 [22.50;83.00] | 32.50 [21.25;54.75] | 0.191 |
| AST(U/L) | 55.00 [34.25;95.50] | 58.00 [41.25;101.00] | 51.50 [26.50;90.75] | 0.108 |
| Immunotherapy |  |  |  | 0.897 |
| Sintilimab | 40(55.6%) | 15(53.6%) | 25(56.8%) |  |
| Camrelizumab | 18(25.0%) | 7(25.0%) | 11(25.0%) |  |
| Tislelizumab | 9(12.5%) | 4(14.3%) | 5(11.4%) |  |
| Cadonilimab | 1(1.4%) | 1(3.6%) | 0(0%) |  |
| Toripalimab | 4(5.6%) | 1(3.6%) | 3(6.8%) |  |
| CombinationTKItreatment |  |  |  | 0.373 |
| Lenvatinib | 44(62.0%) | 15(53.6%) | 29(67.4%) |  |
| Bevacizumab | 12(16.9%) | 7(25.0%) | 5(11.6%) |  |
| Sorafenib | 11(15.5%) | 4(14.3%) | 7(16.3%) |  |
| Regorafenib | 1(1.4%) | 1(3.6%) | 0(0%) |  |
| Apatinib | 3(4.2%) | 1(3.6%) | 2(4.7%) |  |

Continuous variables are presented as mean ± SD or median (interquartile ranges).**Abbreviations:TBIL,** total bilirubin; **AFP,** alpha-fetoprotein; **PT,** prothrombin time; **CRP,** C-reactive protein; **ALT,** alanine aminotransferase; **AST,** aspartate aminotransferase; **TKI,** tyrosine kinase inhibitor.
