# Supplementary material for: Elevated IL-17A level is associated with poor overall survival following immune checkpoint inhibitors combined with targeted therapy in hepatocellular carcinoma with hyperbilirubinemia
Source: Front Immunol. 2026 Apr 7;17:1791538. doi: 10.3389/fimmu.2026.1791538 (PMC13095688; doi:10.3389/fimmu.2026.1791538)
Supplement: Supplementary file 2 [file Table2.docx]

**Table S2. The 36 differentially expressed proteins identified.**

| Regulation | Gene Name | logFC | P.Value | adj.P.Val |
| --- | --- | --- | --- | --- |
| Down | SCF | -1.528406 | 2.50E-09 | 2.30E-07 |
| Up | IL-18R1 | 0.9234526 | 1.09E-08 | 5.01E-07 |
| Up | IL8 | 2.4484605 | 2.73E-08 | 8.38E-07 |
| Up | CCL3 | 1.090246 | 6.41E-07 | 1.47E-05 |
| Up | TGF-alpha | 0.6938906 | 2.95E-06 | 4.85E-05 |
| Up | MCP-1 | 0.7169166 | 3.16E-06 | 4.85E-05 |
| Up | CCL4 | 0.8541971 | 8.36E-06 | 1.10E-04 |
| Up | MMP-10 | 0.881255 | 1.19E-05 | 1.37E-04 |
| Up | TNFSF14 | 0.875147 | 2.80E-05 | 2.86E-04 |
| Up | CD40 | 0.6002888 | 3.45E-05 | 3.18E-04 |
| Up | CXCL6 | 0.8238744 | 5.42E-05 | 4.53E-04 |
| Up | CDCP1 | 0.9949023 | 6.71E-05 | 4.82E-04 |
| Up | CXCL10 | 1.0421 | 6.81E-05 | 4.82E-04 |
| Up | GDNF | 0.7885478 | 9.35E-05 | 5.73E-04 |
| Up | CCL20 | 1.3858468 | 1.01E-04 | 5.83E-04 |
| Up | CX3CL1 | 0.618416 | 1.15E-04 | 6.20E-04 |
| Up | CXCL11 | 0.9470731 | 1.63E-04 | 8.33E-04 |
| Up | uPA | 0.6613995 | 1.87E-04 | 8.61E-04 |
| Up | IL18 | 0.7741503 | 2.07E-04 | 9.05E-04 |
| Up | ADA | 0.5020435 | 2.31E-04 | 9.65E-04 |
| Up | TNF | 0.6703102 | 2.53E-04 | 1.01E-03 |
| Up | LAP TGF-beta-1 | 0.5128875 | 3.27E-04 | 1.26E-03 |
| Up | PD-L1 | 0.6619158 | 4.43E-04 | 1.63E-03 |
| Up | IL6 | 1.2051597 | 7.85E-04 | 2.67E-03 |
| Up | MMP-1 | 0.6944411 | 1.65E-03 | 5.07E-03 |
| Up | HGF | 0.643945 | 2.10E-03 | 6.24E-03 |
| Up | LIF | 0.6625072 | 2.56E-03 | 7.36E-03 |
| Up | IL10 | 0.6179846 | 2.72E-03 | 7.57E-03 |
| Up | CCL19 | 0.7198214 | 2.87E-03 | 7.78E-03 |
| Up | IL-10RA | 0.5373122 | 3.51E-03 | 9.10E-03 |
| Up | FGF-23 | 1.1849166 | 3.94E-03 | 9.54E-03 |
| Up | IL-17A | 0.5565063 | 4.22E-03 | 9.96E-03 |
| Up | IFN-gamma | 0.8126933 | 4.76E-03 | 1.10E-02 |
| Up | CXCL1 | 0.5698558 | 8.19E-03 | 1.75E-02 |
| Up | IL-22 RA1 | 0.5265029 | 1.33E-02 | 2.72E-02 |
| Up | MCP-3 | 0.6090644 | 1.72E-02 | 3.36E-02 |
